# 

**Published:** 1892-04

**Authors:** 


### BOOKS AND PAMPHLETS RECEIVED.

Proceedings of the Colorado Scientific Society. Vol. III. Pt. 3. Denver, 1890.

Bulletin Illinois State Laboratory of Natural History. Vol. III. No. 11–12. Vol. IV. No. 1. Champaigne.

Canadian Record of Science. Vol. IV. No. 8. Montreal, 1891.

Proceedings of the Linnean Society of New South Wales. Vol. VI. Pt. 1. Sydney, 1891.

Verhandlungen der K. K. Zoo. bot. Gesellschaft in wein. Bd. XL. Qu. 1–2. Vienna, 1890.

Ornis. Internationale Zeitschrift fur die gesammte Ornithologie. Jahr. VII. Heft 2–3. Vienna, 1891.

Sitzungberechte der Mathematisch—Physi-Kaleschen Classe der k. b. Akademie der Wessenschaften zu Munchen. Heft 1–4. Munich.

Schriften des Naturwissenschaftlichen Vereins fur Schleswig—Holstein. Bd. VIII.-IX. Kiel.

Ansprach des Prasidenten der K. B. Akademie der Wessenschaften. Dr. Max v. Puttenkofer in der offenthlechen Festsitzung, Nov. 15, 1890. Munich.

Archives Neerlandises des Sciences Exactes et Naturelles. T. XXV. Liv. 3–4. Harlem, 1891.

Bulletin Mensuel Societe Linneeune du Nord de la France. T. X. No. 229–30. Amiens, 1891.

Bulletin de la Societe Zoologique de France. T. XVI. No. 9–10. Paris, 1891.

Bollettino dei Musei di Zoologia ed Anatomia comparata della R. Universita di Torino. Vol. VI. No. 104–11. Turin.

Bulletin de L'Academy Royal de Medicine de Belgique. T. V. No. 11. Bruxelles, 1891.

Bollettino Scientifico. Vol. XIII. No. 3–4. Pavia, 1892.

